# Combined *in-vitro* and on-farm evaluation of commercial disinfectants used against *Brachyspira hyodysenteriae*

**DOI:** 10.1186/s40813-021-00244-9

**Published:** 2022-01-08

**Authors:** Manuel Gómez-García, Héctor Argüello, Lucía Pérez-Pérez, Clara Vega, Héctor Puente, Óscar Mencía-Ares, Pedro Rubio, Ana Carvajal

**Affiliations:** Department of Animal Health, Faculty of Veterinary Medicine, Universidad de León, León, Spain

**Keywords:** Swine dysentery, Disinfection, Cleaning, Internal biosecurity

## Abstract

**Background:**

Swine dysentery (SD) is a severe infectious disease with a relevant impact on pig production usually caused by *Brachyspira hyodysenteriae*, although *B. hampsonii* causes an identical clinical picture. SD control relies on antimicrobials, good management practices and strict biosecurity with cleaning and disinfection as crucial tools to avoid the pathogen transmission. This study evaluates the *in-vitro* efficacy of an array of commercial disinfectants against a collection of *B. hyodysenteriae* isolates using broth tests. The efficacy of cleaning and disinfection protocols was also evaluated on two farms with endemic SD using surface swabs collected in emptied pens before and after cleaning and disinfection procedures, using both real-time PCR and bacterial microbiological culture.

**Results:**

Most of the commercial disinfectants evaluated were effective against all *B.* *hyodysenteriae* isolates tested, with a reduction of more than 5.00 log_10_ CFU/mL (bactericidal efficacy of 99.999%). However, some isolates exhibited reduced susceptibility to Virkon-S and Limoseptic disinfectants. The evaluation of cleaning and disinfection protocols on farms with SD outbreaks showed that approximately half the pens tested (n = 25) were positive by real-time PCR after pigs removal (mean *B.* *hyodysenteriae* counts 5.72 ± 1.04 log_10_ CFU/mL) while almost 20% of the pens remained positive after cleaning (n = 7) and disinfection (n = 5) procedures although with significantly lower, mean estimates (4.31 ± 0.43 log_10_ CFU/mL and 4.01 ± 0.55 log_10_ CFU/mL, respectively).

**Conclusions:**

These results show the efficacy of disinfectants against *B. hyodysenteriae* but also stress the need to implement adequately the cleaning and disinfection protocols on pig farms and review and revise their efficiency periodically.

## Background

Swine dysentery (SD) is a severe mucohaemorhagic enteric disease, which causes important losses in the pig industry due to mortality and sub-optimal performance [1, 2]. The disease is more frequently observed in the growing and finishing stages [3]. The classical etiological agent is *Brachyspira hyodysenteriae,* a Gram-negative, motile, helically coiled, beta-haemolytic and anaerobic bacteria [4]. *Brachyspira hyodysenteriae* colonizes the lumen and crypts of the porcine caecum and colon causing mucohemorrhagic diarrhoea [1]. *Brachyspira hampsonii* infection is mainly confined to North America and can cause the same clinical signs [5, 6].The transmission by the faecal-oral route occurs by direct contact through the introduction of infected animals into uninfected herds [7] and by indirect contact with contaminated surfaces, where the pathogen is capable of surviving under favourable conditions such as organic matter, humidity and darkness [3].

Limitations in disease treatment, linked to the emergence of strains with reduced susceptibility to antibiotics or the lack of commercial vaccines, highlight the relevance of other strategies in SD prevention and control [8–10]. High standards in biosecurity are crucial in prevention and amelioration of diseases in pig production [11, 12] and management strategies such as all-in /all-out (AI/AO) attempt to be effective firewalls to prevent the transmission of diseases such as SD. Undoubtedly the effectiveness of AI/AO depends on the efficacy of cleaning and disinfection protocols put in place [2, 3, 11].

Disinfectant choice depends on factors such as microorganism spectrum, surfaces to be treated, applicable temperature range, toxicity or economic constraints [13]. Despite the theoretical efficacy of disinfectants, their misuse favours the emergence and spread of disinfectant tolerance [14] by the selection of resistant clones/strains and the horizontal spread of disinfectant resistance genes [15]. Field studies also reveal the need to implement efficient protocols, which remove the pathogens from the environment successfully [16]. Despite the environmental component in SD epidemiology, there are no studies which particularly combine the assessment of the *in-vitro* susceptibility of *B. hyodysenteriae* to disinfectants and the efficacy of these agents under the usual cleaning and disinfection protocols implemented in field conditions [2, 17, 18]. With this aim in mind, this study evaluates the *in-vitro* efficacy of an array of different commercial disinfectants against a collection of ten field isolates of *B.* *hyodysenteriae* and the efficacy of hygiene protocols, under field conditions, on farms with SD.

## Results

### *In-vitro* efficacy of disinfectants against *B. hyodysenteriae*

The results of disinfectants activity against *B. hyodysenteriae* are shown in Table 1. Mean *B. hyodysenteriae* counts in control tests (without disinfectant) was 7.6 ± 0.2 log_10_ colony forming units (CFU)/mL and we observed that disinfectants activity was not inhibited by the interfering substance. All disinfectants, except for Virkon-S and Limoseptic, were capable of inhibiting the *B. hyodysenteriae* viability completely (reduction of more than 5.00 log_10_ CFU/mL or 99.999% efficacy) with no differences among the isolates tested. However, reduced efficacy was observed with VIRKON-S against the isolate IT-40 (average reduction of 2.9 ± 0.3 log_10_ CFU/mL), IT-67 (2.2 ± 0.8 log_10_ CFU/mL) and IT-85 (3.1 ± 0.9 log_10_ CFU/mL) and with Limoseptic against IT-45 (3.9 ± 0.6 log_10_ CFU/mL).

**Table 1:** Surviving population of each *B. hyodysenteriae* isolate after exposure to the disinfectant tested

	Mean counts (log_10_ CFU/mL) and standard deviation ( ±)										
Disinfectant	Isolate										QC strain ^b^
	IT-1	IT-18	IT-39	IT-40	IT-45	IT-48	IT-67	IT-68	IT-83	IT-85	B204
ET-70%^a^	0.0 ± 0.0	0.0 ± 0.0	0.0 ± 0.0	0.0 ± 0.0	0.0 ± 0.0	0.0 ± 0.0	0.0 ± 0.0	0.0 ± 0.0	0.0 ± 0.0	0.0 ± 0.0	0.0 ± 0.0
Virkon-S	0.0 ± 0.0	0.0 ± 0.0	0.0 ± 0.0	**4.7 ± 0.3**	0.0 ± 0.0	0.0 ± 0.0	**5.0 ± 0.4**	0.0 ± 0.0	0.0 ± 0.0	**4.8 ± 0.6**	0.0 ± 0.0
CR-36	0.0 ± 0.0	0.0 ± 0.0	0.0 ± 0.0	0.0 ± 0.0	0.0 ± 0.0	0.0 ± 0.0	0.0 ± 0.0	0.0 ± 0.0	0.0 ± 0.0	0.0 ± 0.0	0.0 ± 0.0
Yodermin	0.0 ± 0.0	0.0 ± 0.0	0.0 ± 0.0	0.0 ± 0.0	0.0 ± 0.0	0.0 ± 0.0	0.0 ± 0.0	0.0 ± 0.0	0.0 ± 0.0	0.0 ± 0.0	0.0 ± 0.0
Poliformo	0.0 ± 0.0	0.0 ± 0.0	0.0 ± 0.0	0.0 ± 0.0	0.0 ± 0.0	0.0 ± 0.0	0.0 ± 0.0	0.0 ± 0.0	0.0 ± 0.0	0.0 ± 0.0	0.0 ± 0.0
Limoseptic	0.0 ± 0.0	0.0 ± 0.0	0.0 ± 0.0	0.0 ± 0.0	**3.5 ± 0.5**	0.0 ± 0.0	0.0 ± 0.0	0.0 ± 0.0	0.0 ± 0.0	0.0 ± 0.0	0.0 ± 0.0
MS Megades Oxy	0.0 ± 0.0	0.0 ± 0.0	0.0 ± 0.0	0.0 ± 0.0	0.0 ± 0.0	0.0 ± 0.0	0.0 ± 0.0	0.0 ± 0.0	0.0 ± 0.0	0.0 ± 0.0	0.0 ± 0.0
MS Megades Novo	0.0 ± 0.0	0.0 ± 0.0	0.0 ± 0.0	0.0 ± 0.0	0.0 ± 0.0	0.0 ± 0.0	0.0 ± 0.0	0.0 ± 0.0	0.0 ± 0.0	0.0 ± 0.0	0.0 ± 0.0
Control (without disinfectant)	**7.8 ± 0.3**	**7.7 ± 0.6**	**7.4 ± 0.3**	**7.5 ± 0.0**	**7.5 ± 0.1**	**7.5 ± 0.5**	**7.5 ± 0.6**	**7.4 ± 1.0**	**7.6 ± 1.0**	**8.0 ± 0.4**	**8.1 ± 0.1**

^a^ Positive control

^b^ Quality control strain

### On-farm efficacy of cleaning and disinfection protocols

As shown in Table 2, detection of *B. hyodysenteriae* by real-time PCR revealed that 44.6% of the pens tested (n = 56) were positive to *B.* *hyodysenteriae* after being emptied of pigs and before they were cleaned (BC). In contrast, only 12.5% and 8.9% of the pens tested were positive after cleaning (AC) and after disinfection procedures (AD), respectively. Similar percentages were obtained in the analysis of results for each farm, despite the number of pens tested on farm B (n = 48) was considerably higher than on farm A (n = 8).

**Table 2:** Results of *B. hyodysenteriae* detection by real-time PCR in the pens sampled

	Before cleaning (BC) Sampled pens			After cleaning and before disinfection (AC) Sampled pens			After disinfection (AD) Sampled pens		
	Total	Positive	%	Total	Positive	%	Total	Positive	%
Combined results	56	25	44.6	25	7	28.0	25	5	20.0
Farm A ^#^	8	6	75.0	6	1	16.7	6	1	16.7
Farm B ^#^	48	19	39.6	19	6	31.6	19	4	21.1

^#^ A two-step protocol including power washing and disinfection with Hypred Force 7 was carried out on Farm A while Farm B cleaning and disinfection protocol included pre-soaking with cold water and detergent, power washing with cold water and final disinfection with MS Megades Oxy

When only *B.* *hyodysenteriae-*positive pens BC (n = 25) were included in the analysis, the percentage of pens which remained positive AC and AD increased to 28.0% and 20.0%, respectively. The proportion of positive pens on farm A both AC and AD was lower than on farm B (Table 2), although results did not reach statistical significance (*p* > 0.05).

**Fig. 1:**
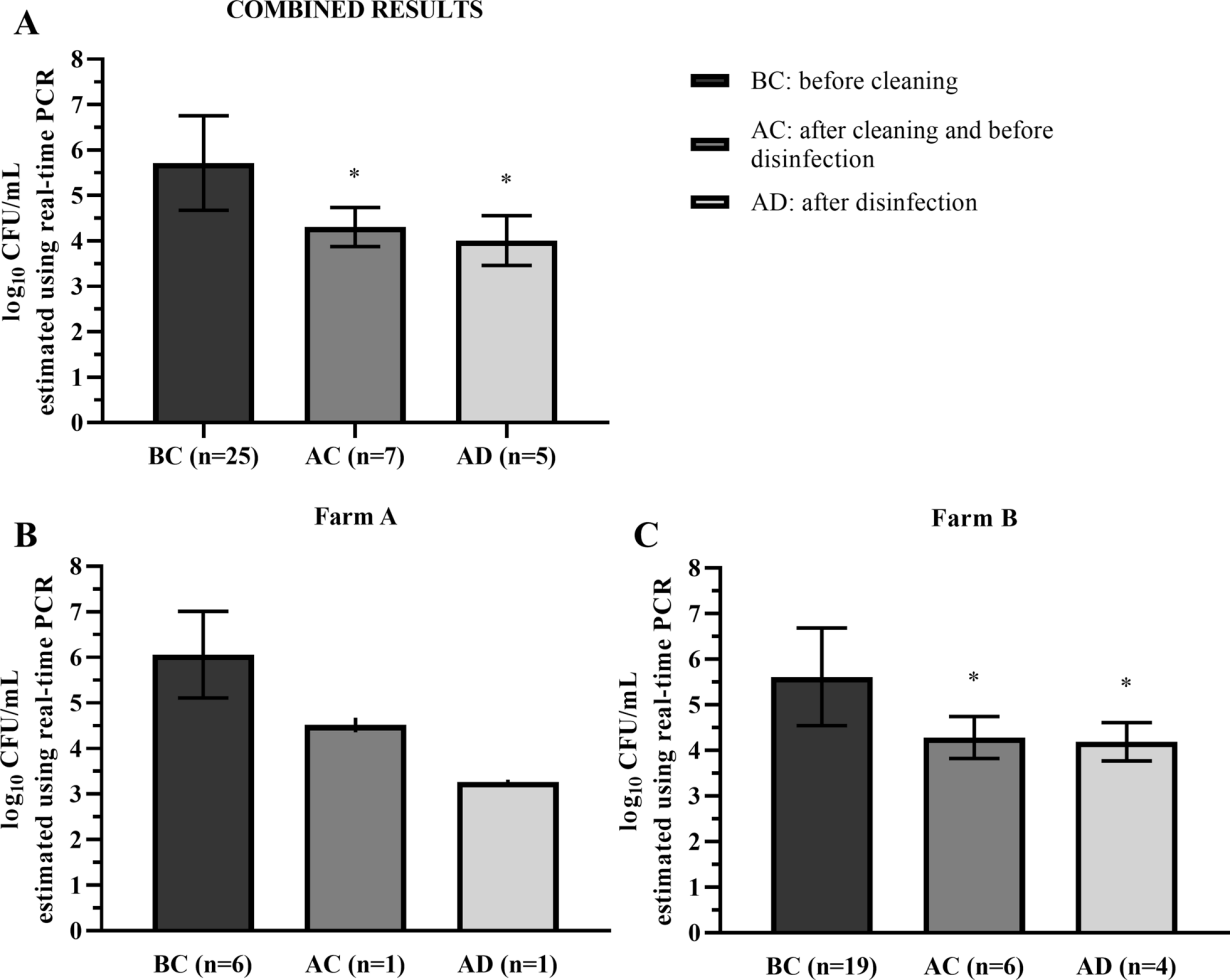
Mean *B. hyodysenteriae* counts estimated using real-time PCR in the pens sampled. Mean values ± standard deviations of log_10_ CFU/mL estimated using real-time PCR in all *B. hyodysenteriae*-positive pens before cleaning (BC) included in this study (**A**) as well as on farm A (**B**) and farm B (**C**). A two-step protocol including power washing and disinfection with Hypred Force 7 was carried out on Farm A while Farm B cleaning and disinfection protocol included pre-soaking with cold water and detergent, power washing with cold water and final disinfection with MS Megades Oxy. * Denotes statistically significant differences when compared with the *B. hyodysenteriae* counts before cleaning protocol (*p* < 0.05). Statistical analysis could not be made for Farm A because only one *B. hyodysenteriae*-positive pen was identified after cleaning and before disinfection (AC) and after disinfection (AD)

By using real-time PCR, we also aimed at establishing the *B. hyodysenteriae* counts (log_10_ CFU/mL) in positive samples (Fig. 1). Estimated mean counts of the pathogen in positive swabs from emptied pens were 5.7 ± 1.0 log_10_ CFU/mL. Mean counts obtained AC (4.3 ± 0.4 log_10_ CFU/mL) and AD (4.0 ± 0.6 log_10_ CFU/mL) were significantly lower (*p* < 0.05) than *B. hyodysenteriae* values BC (Fig. 1A). However, we did not observe any significant variation of log_10_ CFU/mL between cleaning and disinfection protocols (*p* > 0.05).

On farm A (Fig. 1B), the only *B. hyodysenteriae*-positive pen AC showed a lower value of *B. hyodysenteriae* counts (4.5 ± 0.2 log_10_ CFU/mL) than the values obtained BC (6.1 ± 0.9 log_10_ CFU/mL) and the bacterial load clearly decreased after disinfection with Hyper Force 7 (3.3 ± 0.1 log_10_ CFU/mL). Farm B (Fig. 1C), using MS Megades Oxy, showed similar *B. hyodysenteriae* estimations. In detail, the means of log_10_ CFU/mL estimated BC, AC and AD were 5.6 ± 1.1, 4.3 ± 0.5 and 4.2 ± 0.4, respectively.

*Brachyspira hyodysenteriae* could only be isolated from a single environmental sample, which was obtained in the first sampling (BC) on farm B. The quantification of *B. hyodysenteriae* by real-time PCR revealed that the bacterial load in this sample was the highest estimated in our study (7.47 ± 0.01 log_10_ CFU/mL). The bacteria did not grow in any other environmental sample, neither positive nor negatives, in the real-time PCR analyses.

## Discussion

Preventive medicine is essential to combat livestock infectious diseases in the post-antibiotic era and biosecurity has gained relevance in difficult-to-control diseases such as SD [19]. Within biosecurity schemes, implementation of effective cleaning and disinfection protocols are mandatory as part of those strategies, which aim at breaking the disease transmission among batches [11]. This fact is supported by observational studies such as the one carried out by Neirynck et al. [2], who only found successful eradication programmes for *B. hyodysenteriae* on the four farms, which implemented properly cleaning and disinfection procedures, among other biosecurity measures. This efficacy relies on the selection of the most appropriate compounds [14, 15] and their application through effective protocols. In this way, there exist only a few research studies focusing on the susceptibility of *B. hyodysenteriae* to disinfectants and the efficacy of current cleaning and disinfection protocols on farms with endemic SD.

The results of our study under laboratory conditions confirmed the antibacterial activity of most commercial disinfectants tested against field isolates of *B.* *hyodysenteriae.* Earlier studies evaluating the *in-vitro* activity of disinfectants from different chemical groups against a type strain and a field *B. hyodysenteriae* isolate [20] or a collection of seven *B. pilosicoli* [21] converged in the conclusion that disinfectants were effective against *Brachyspira* spp*.*, even at concentrations lower than that recommended by manufacturers. Our results show that following the European Norm (EN) 1656:2009, most disinfectants can be considered as effective products under the assay conditions, as they reduced 99.999% of bacteria in suspension after 30 min of contact. However, the fact that a few numbers of isolates experienced lower sensitivity against Virkon-S, a peroxygen disinfectant and Limoseptic, an ammonium derivate shows that slight strain-dependent variations can occur. It is worth mentioning that two of the four isolates exhibiting higher tolerance to Virkon-S and Limoseptic disinfectants also showed high antibiotic MIC values. The increase and the potential spread of isolates with reduced susceptibility to antibiotics and disinfectants limits two of the most efficient strategies in SD control and highlights the relevance of early detection of these problematic strains.

Sterile faeces are usually included in the *in-vitro* evaluation of disinfectants. Despite the fact that it has been shown than faeces can increase the MIC of a disinfectant needed to inhibit the growth of *Brachyspira* spp. [20, 21], our results show, as in other studies [22], that the presence of faeces did not affect the *in-vitro* activity of the compounds at the recommended concentrations. However, we must point out that under field conditions, large amounts of faeces or organic matter can inhibit disinfectant activity [23].

We further researched the efficacy of disinfectants in practice by monitoring routine cleaning and disinfection protocols on two farms with endemic SD. Environmental detection of *B. hyodysenteriae* in pens from grower and finisher pig batches with clinical outbreaks of SD showed the presence of relevant concentrations of the pathogen in at least half of the pens tested using real-time PCR. Despite the fact that this molecular diagnostic technique does not differentiate viable from non-viable *B. hyodysenteriae*, the DNA mean values reached (5.72 ± 1.04 log_10_ CFU/mL) are not far from the experimental dose in SD challenges [24] and provide an idea of the load of pathogen in emptied pens after animals have been moved. However, the parallel culture of fresh samples did not support our PCR results. *Brachyspira hyodysenteriae* is a fastidious anaerobe and factors such as antimicrobial treatments, time lapse between shedding and sample collection and/or dilution of pathogen concentration by dejections may hamper its further laboratory isolation, an experience shared with a previous environmental study of *B. hyodysenteriae* viability [18]. The fact that subsequent batches of pigs experienced SD on both farms points out the viability of the *B. hyodysenteriae* detected in the pens.

Surface sampling of pens AC and AD highlighted the presence of the pathogen in almost a third of the positive pens, with no improvement of the disinfection step compared to power washing (farm A) or washing with detergent (farm B), a result which contrasts with our *in-vitro* results and shows the failure of the protocols put in place, particularly on farm B, where the visual inspection of pens evidenced dirty areas after finishing the cleaning protocol (data not shown). Despite that the cleaning and disinfection protocols reduced 80% of initial *B. hyodysenteriae*-positive pens, viable *B. hyodysenteriae* remaining on pen surfaces, feeders, corridors or equipment are potential source of infection for the forthcoming batches of animals [3, 20]. *Brachyspira hyodysenteriae* quantification estimates in pens remaining positive revealed a log reduction of counts after cleaning (1.40 log_10_ CFU/mL), with no further improvement AD. A similar reduction in *Enterobacteriaceae* counts was achieved by a similar protocol including high-pressure washing in the lairage environment of a pig abattoir [16]. Disinfection and drying also removed *Salmonella* and reduced *Enterobacteriaceae* counts in the aforementioned study. We did not observe such benefit in *B. hyodysenteriae* estimates, with a reduction after disinfectant application on farm A positive pen (1.79 log_10_ CFU/mL) and had no effect on pens from farm B either.

## Methods

### Bacterial strains and disinfectants

A set of ten isolates of *B.* *hyodysenteriae* kept in the bacteriological collection from the DIGESPORC research group at the University of León was used in this study. The isolates were recovered from the diagnosis submissions from diarrhoea outbreaks on Spanish swine farms between January 2018 and December 2019. Table 3 gives the list of isolates used and their antimicrobial profile, determined using a broth microdilution procedure as previously described [25] using VetMIC Brachy antibiotic panels (SVA, Sweden).

**Table 3:** MIC values (µg/mL) of six antimicrobial agents obtained against the 10 *B.* *hyodysenteriae* isolates tested

Antimicrobial agent and concentration range (μg/mL)	MIC (µg/mL)									
	Isolate									
	IT-1	IT-18	IT-39	IT-40	IT-45	IT-48	IT-67	IT-68	IT-83	IT-85
Tiamulin (0.063–8)	8	0.125	1	> 8	> 8	4	> 8	1	0.125	0.5
Valnemulin (0.031–4)	> 4	< 0.031	0.5	4	0.25	4	> 4	1	< 0.031	0.5
Doxycycline (0.125–16)	1	1	0.25	2	16	1	16	2	1	0.5
Tylvalosin (0.25–32)	> 32	4	0.5	> 32	1	2	16	8	8	1
Lincomycin (0.5–64)	32	32	32	> 64	> 64	> 64	32	32	32	16
Tylosin (2–128)	> 128	> 128	4	> 128	> 128	> 128	8	> 128	> 128	> 128

We collected information on disinfectants commonly used from nearby farms within our region, finally selecting eight commercial disinfectants and ethanol (used as effective disinfectant control). Details of disinfectants composition and use conditions are detailed in Table 4.

**Table 4:** Composition and final concentration of the working solution of the disinfectants tested

Disinfectant	Main bactericidal components and concentration	Working concentration
ET-70% (alcohol)	70% ethanol	100%^a^
Virkon-S (peroxygen compound)	49.7% Pentapotassium bis(peroxymonosulphate) bis(sulphate) and organic acids	1%
CR-36 (alcohol and quaternary ammonium)	0.256% 2-bromo-2-nitro-1,3-propanediol	100% ^a^
Yodermin (povidone-iodine)	10% Polyvinylpyrrolidone iodine (equal to 1% available iodine)	100% ^a^
Poliformo (Phenol)	10% p-chloro-m-cresol	2%
Limoseptic (glutaraldehyde and quaternary ammonium)	5% glutaraldehyde and 4.5% didecyldimethylammonium chloride	1%
MS Megades Novo (glutaraldehyde and quaternary ammonium)	15% glutaraldehyde and 10% quaternary ammonium	0.75%
MS Megades Oxy (peroxygen compound and peracetic acid)	7.8% hydrogen peroxide and 2.4%peracetic acid	0.5%
Hypred Force 7 * (glutaraldehyde and quaternary ammonium)	13% glutaraldehyde, 1.5% didecyldimethylammonium chloride and 8% quaternary ammonium compounds, benzylalkyldimethyl and chlorides	2%

* HYPRED FORCE 7 was only tested under field conditions

^a^ not diluted

### *In-vitro* efficacy of disinfectant against *B. hyodysenteriae*

The evaluation of bactericidal activity of disinfectants was carried out using a dilution-neutralisation method according to the recommendations of EN 1656:2009. In brief, 100 µL of a bacterial suspension (approximately 10^7^ CFU/mL) and 100 µL of sterile faeces (121 °C, 15 min in autoclave) as interfering substance were added to 800 µL of each disinfectant diluted in hard water to obtain the concentration recommended by the manufacturers (Table 4). After 30 min incubation at 10 °C, 100 µL of the mixture was mixed with 100 µL of sterile water and 800 µL of Dey-Engley neutralising broth (BD Difco, United States) and again incubated for 5 min at 20 °C.

Estimation of the survival population was performed by plating ten-fold serial dilutions in phosphate-buffered saline (PBS, pH 7.4) into Trypticase Soy Agar (TSA) supplemented with 5% sheep blood (Oxoid, Spain). Finally, plates were incubated in a bug box anaerobic workstation (Baker Ruskinn, United States) with an oxygen-free anaerobic gas mixture (80% N_2,_ 10% H_2_ and 10% CO_2_) at 39 °C for four-six days, the period after which the surviving population (log_10_ CFU/mL) was estimated. Each test was carried out in duplicate using a fresh culture suspension. *B. hyodysenteriae* reference strain B204 (ATCC 31212) and ethanol (ET-70%) were also included as a quality control strain and disinfectant control, respectively.

Finally, the viability of *B. hyodysenteriae* in sterile faeces and neutralising broth was confirmed and the neutralising capacity of the Dey-Engley neutralising broth against each disinfectant was also checked before running the experiment.

### On-farm efficacy of cleaning and disinfection protocols

Field efficacy of cleaning and disinfection protocols was evaluated on two commercial pig farms with endemic SD. A two-step protocol including power washing and disinfection with Hypred Force 7 was carried out on Farm A while Farm B cleaning and disinfection protocol included pre-soaking with cold water and detergent, power washing with cold water and final disinfection with MS Megades Oxy. Farm characteristics, the specific soapy detergent and disinfectant used and number of pens per farm are detailed in Table 5.

**Table 5:** Details of farms participating in the evaluation of the cleaning and disinfection against *B.* *hyodysenteriae*

FARM	Type of swine production	N^o^ pens initially included	Floor type	Weight of animals	Soapy detergent	Disinfectant
A	Finishing unit	8	Part slatted (concrete)	72 kg	Not used	Hypred Force 7
B	Farrow to grower	48	Fully slatted (plastic)	20 kg	MS TopFoam LC Alk	MS Megades Oxy

Following the same procedure, four squares of each pen evaluated (approximately 25 cm^2^ each) were swabbed BC and AC using a sterile gauze per pen placed into 50 mL of PBS (pH 7.4) and AD using a cellulose sponge per pen supplied in sterile bags with 10 mL of neutralising buffer (3 M HydraSponge, United States). In both AC and BD sampling, the pens were visually inspected before sampling and dirty areas were selected for swabbing if detected. Samples were handled aseptically under cooling conditions and processed within 24 h in the laboratory.

### *Brachyspira hyodysenteriae* detection and quantification by real-time PCR

Gauzes and sponges embedded with PBS or neutralising buffer were placed into sterile bags and homogenized using a stomacher (Seward 400, UK) for 5 min. From each sample, a final volume of 500 µL were further used to extract DNA using GeneMATRIX Stool DNA Purification Kit (EurX, Poland), following the manufacturer’s recommendations.

A species-specific real-time PCR assay was carried out to detect the presence of *B. hyodysenteriae* using the primers, probes and the cycling conditions previously described [26]. Each reaction mixture (20 µL final volume) contained 8 µL of Maxima Probe real-time PCR Master Mix 2X (Thermo Scientific, Stockholm, Sverige), 0.3 µL of 10 µM each primer, 0.15 µL of 10 µM Taq-Man probe, 0.12 µL of Rox (diluted 1:10 in nuclease-free water, Thermo Scientific, Sweden), 9.28 µL of nuclease-free water and 2 µL of extracted DNA. The assay was carried out in a QuantStudio 1 thermal cycler (Applied Biosystems, United States) and the *B. hyodysenteriae* counts in targeted samples was estimated using a standard curve which was prepared using ten-fold serial dilutions of a *B. hyodysenteriae* B204 pure culture (initial load 8 log_10_ CFU/mL and range 8 to 2 log_10_ CFU/mL). DNA extraction of standard curve broth was carried out as described above for environmental samples. The detection limit was defined by the linear portion of the standard curve and was set at 3 log_10_ CFU/mL (Cycle threshold value of 35.7). Each DNA sample was analysed in duplicate.

### *Brachyspira hyodysenteriae* detection and isolation in selective media

In parallel to real-time PCR detection and quantification of *B. hyodysenteriae*, samples were cultured to detect, isolate and purify *B. hyodysenteriae* following the methodology described [27]. In brief, TSA plates (Scharlab, Spain) supplemented with 5% ovine blood (Oxoid, Spain) and antibiotics (400 µg/mL spectinomycin, 8 µg/mL colistin and 20 µg/mL vancomycin, Sigma-Aldrich, United States) was used for primary isolation. Suspected positive samples showing strong β-haemolysis and spirochaetes in phase-contrast microscopy were confirmed using species-specific PCR based on the *tlyA* gene [28]. TSA supplemented with 5% ovine blood agar (Oxoid, Spain) was further used for subsequent subcultures until a pure growth was confirmed by phase-contrast microscopy. All cultures were carried out under optimal conditions for the growth of *B. hyodysenteriae* previously described.

### Statistical analysis

The analysis was carried out with IBM SPSS Statistics version 26 at the 5% significance level. The *B.* *hyodysenteriae* counts (log_10_ CFU/mL) estimated by real-time PCR were tested for normality (Kolmogorov–Smirnov test) and statistical differences among different samplings (BC, AC and AD) were evaluated using the ANOVA test. Differences in proportions of positive pens among farm A and farm B were checked using chi-square or Fisher’s exact tests where appropriate.

## Conclusions

This study shows the susceptibility of *B. hyodysenteriae* field isolates to currently used commercial disinfectants under laboratory conditions. However, in practice, the cleaning and disinfection protocols evaluated in this study frequently failed to remove *B. hyodysenteriae,* probably, as a consequence of deficient pen faecal removal. Using real-time PCR, we showed a high load of the pathogen in emptied pens and a low reduction achieved using protocols tested. The result is a warning for farms with endemic SD that the cleaning and disinfection protocols in use should be evaluated.

## Data Availability

The authors declare that they did not apply new software and databases.

## Abbreviations

SD: Swine dysentery
AI/AO: All-in/all-out
EN: European Norm
CFU: Colony forming units
BC: Before cleaning
AC: After cleaning and before disinfection
AD: After disinfection
PBS: Phosphate-buffered saline
TSA: Trypticase Soy Agar
ATCC: American Type Culture Collection
